# Children Are Exposed to Fecal Contamination via Multiple Interconnected Pathways: A Network Model for Exposure Assessment

**DOI:** 10.1111/risa.13146

**Published:** 2018-07-27

**Authors:** Yuke Wang, Christine L. Moe, Peter F. M. Teunis

**Affiliations:** ^1^ Center of Global Safe Water, Sanitation, and Hygiene, Hubert Department of Global Health Rollins School of Public Health, Emory University Atlanta GA USA

**Keywords:** Exposure assessment, fecal contamination, infectious disease, multipathway, network modeling

## Abstract

In recent decades, quantitative microbial risk assessment (QMRA) has been widely used to assess exposure to fecal microbes and associated health risks. In this study, a multipathway exposure assessment model was developed to evaluate exposure to fecal microbes for children under 5 in highly contaminated urban environments. Children had contact with various environmental compartments. The contamination levels of these compartments were estimated from fecal indicator counts in the environmental samples. Structured observations of child behavior (including activities, locations, and time) were used to model behavioral sequences as a dynamic network. The exposure model combines behavior sequences with environmental contamination, using additional exposure factors when needed, to estimate the number of fecal microbes transferred from environmental sources to human oral ingestion. As fecal exposure in a highly contaminated urban environment consists of contributions from multiple pathways, it is imperative to study their relative importance. The model helps us better understand the characteristics of the exposure pathways that may be driven by variation in contamination and by variable behavior, like hygiene and high‐risk activities. Importantly, the model also allows prediction of the quantitative effects of an intervention—the expected reduction in exposure due to infrastructural or behavioral changes—by means of scenario studies. Based on experience with this exposure model, we make specific recommendations for additional studies of child behavior and exposure factors in order to fill critical information gaps and improve the model structure and assumptions.

## 1. INTRODUCTION

Exposure to fecal contamination has a negative impact on child health, growth, and development. A short‐term effect of exposure to fecal pathogens is diarrheal disease, which is a leading cause of mortality and morbidity in children under 5 in low‐income countries (World Health Organization, 2009). Long‐term exposure to fecal contamination increases the risk of environmental enteric dysfunction, malnutrition, and stunting (Humphrey, 2009; Jiang, Tofail, Ma, Haque, Kirkpatrick, Nelson, & Petri, 2017; Mbuya & Humphrey, 2016).

When children live in a highly contaminated environment, multiple sources contribute to exposure to fecal microbes simultaneously and dynamically. In 1958, the F‐diagram was introduced by Wagner and Lanoix to explain multiple pathways and barriers for disease transmission (Wagner & Lanoix, 1958). This conceptual diagram illustrates how fecal microbes “travel” from sources to human oral ingestion, and how different sanitation and hygiene interventions influence transmission pathways for enteric disease.

Methods for quantitative microbial risk assessment (QMRA) have become increasingly popular for assessing exposure to fecal microbes, and predicting the associated health risks (Haas, Rose, & Gerba, 1999) for waterborne exposure (Barker, Amoah, & Drechsel, 2014; McBride, Stott, Miller, Bambic, & Wuertz, 2013) in low‐income country settings (Labite et al., 2010; Machdar, van der Steen, Raschid‐Sally, & Lens, 2013). However, many QMRA studies only assessed a specific pathway that was considered the riskiest pathway based on prior information or expert opinions (McBride et al., 2013). Some studies assessed more than one pathway but assumed independence between pathways (Barker et al., 2014; Machdar et al., 2013). The health risks associated with exposure to a network of pathways that have multiple connections, as illustrated by the F‐diagram, have not been studied quantitatively.

The present study was designed to build an agent‐based model (An, 2012; Bonabeau, 2002; Eubank et al., 2004) to assess multiple fecal exposure pathways and their interactions using a network structure approach. The model combines levels of fecal contamination in the environment, behavior sequences for contact with contaminated environments, and microbe transfer characteristics. Multiple pathways may interact by sharing the same vehicle for transmission of fecal contamination, often the hands of the subject. The numbers of fecal microbes on ingested media, food, or mouthed objects (including hands) result from a prior history of microbe transfers, often controlled by the behavior of the exposed subject. For that reason, the model presented here is based on a quantitative model of child behavior (Teunis, Reese, Null, Yakubu, & Moe, 2016). The present article documents the construction of a multipathway exposure model, provides details of the simulations of microbe transfer for all pathways that were included, and explains the methods used for estimating parameters from the microbial data that were collected. An overview of the conceptual framework and major results has been published (Wang et al., 2017).

## 2. MODEL COMPONENTS

The main entry point for exposure to enteric pathogens is via oral ingestion. Oral exposure can be through direct swallowing of contaminated matter, like drinking water or eating food. Exposure may also result from indirect contact with contaminated environments, e.g., by mouthing contaminated hands. An exposure pathway is defined as a link between a source of fecal contamination and a destination (a sink), here the mouth of the exposed subject. Environmental sources may be any contaminated food and drink, soil, and contaminated surfaces, including highly contaminated compartments like open drains or septage. In a contaminated environment, people usually are exposed to multiple pathways simultaneously, with hands serving as intermediate (vehicle) in many pathways. Contacts are highly dynamic, and the order in which they occur can determine their influence on exposure. Suppose a child touches a dirty floor and then eats food; his/her hands may be cleaned before or after eating. In the first case, the risk of exposure is expected to be lower than in the second case. Therefore, the behavior model not only estimates the duration or frequencies of activities, but also the order in which they occur (Teunis et al., 2016).

The behavior that drives exposure to fecal contamination is variable. The levels of contamination in, or on, the contacted media are also highly variable. Concurrent with the behavioral studies, a comprehensive sampling program of environmental sources of fecal contamination was conducted in the same neighborhoods in Accra, Ghana (Robb et al., 2017). The exposure assessment in this article is based on analysis of *Escherichia coli* concentrations in samples of food, water, and other environmental compartments collected in the SaniPath study. In addition to the data used in the behavior and environmental concentration models, a third major source of information was exposure factors (e.g., amounts of microbes ingested, or transferred upon contact with contaminated media). Some of the exposure factors were based on data reported in the scientific literature, and not on data collected in the SaniPath study.

### 2.1. Competing Hazards Model of Child Behavior

**Figure 1:**
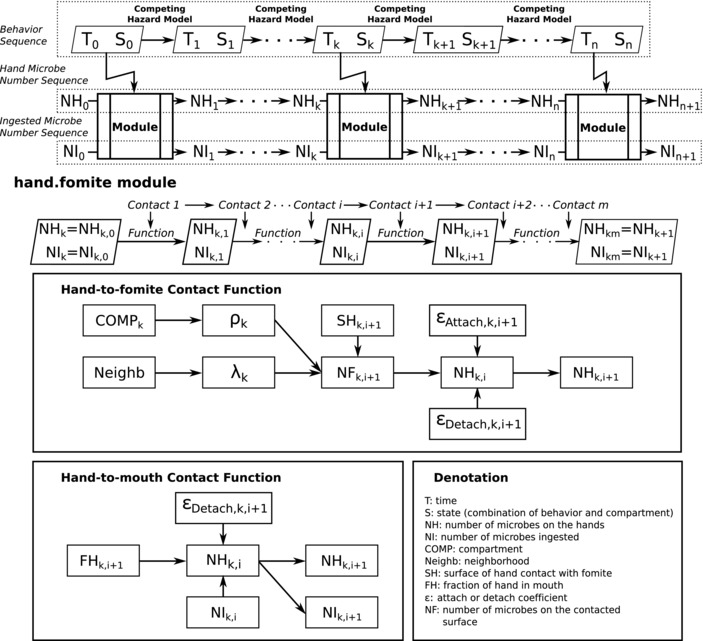
Exposure model structure. Time is denoted as “*T*.” To fully describe the behavior of a child, one needs to define what behavior is occurring, and where this happens. State (S) is a combination of behavior (BEH) and compartment (COMP). The competing hazard model will generate the next time (T) and state (S) until $T_{n}-T_{0}>14$ hours. Behavior sequence, defined by Teunis et al. (2016), consists of a sequence of T and a sequence of S. The number of microbes on hands (NH) and the number of microbes ingested (NI) will also be sequences generated by exposure modules selected based on current duration (difference in time) and state. The hand.fomite module shows how hand contamination sequence *NH* and ingestion sequence *NI* are generated (${\mathrm{NH}}_{k}->{\mathrm{NH}}_{k+1}$, ${\mathrm{NI}}_{k}->{\mathrm{NI}}_{k+1}$).

Child behavior was simulated as a sequence of “states” (Fig. 1), designated as a combination of any of six activities (playing/sitting, sleeping, handwashing, bathing, defecating, and eating) and any of five compartments where these activities occur (dirt floor, improved [concrete] floor, off‐ground, stagnant water/trash area, and open drain). The behavior of any child may then be thought of as traveling on a network, with states as nodes, and transitions between states as edges, and the rates with which transitions occur as edge weights. Thus, the behaviors of children can be represented in a directed, weighted network. From this network structure, the observed behaviors were analyzed with a competing hazards model, which estimates the rates of transitions as a set of motivations competing for the next state. The longer a child is observed in any state, the higher that state is weighted in the survival model. Transition rates appeared to depend on the present state, but also on the state directly preceding the current state. The details of this behavioral model have been described by Teunis et al. (2016)

With these estimated transition rates, a simulation model was built that allowed the generation of sequences of behaviors. Each state in these sequences is occupied with a random duration, selected from competing hazards that were estimated from over 500 hours of structured observation data (Teunis et al., 2016). Thus, a random “day in the life of a child” is simulated, where any behavior at any time is completely known. The behavioral parameters (hazard rates) were estimated by neighborhood (Alaja, Bukom, Old Fadama, and Shiabu) and age category (zero to one, one to two, and two to five years old).

### 2.2. Mixture Model for Environmental Contamination

A total of 1,855 environmental samples were collected in four neighborhoods in Accra, Ghana, including liquid samples (tap water, household stored water, drain water, ocean water, flood water, and irrigation water), solid samples (soil, sand, sediment, vendor food, and uncooked produce), and surface swabs (concrete floor, off‐ground surface, and public latrine surface), as potential sources of exposure for children.

Contamination in environmental samples was characterized by *E. coli* as a fecal microbial indicator, detected by membrane filtration, and resulting in counted numbers of colony‐forming units (CFUs, in replicate) and corresponding equivalent volumes for each observation. All samples were tested in multiple dilutions. Solid samples and surface swabs were first suspended in standardized volumes of buffer (Sanipath website, 2018). It was common to observe considerable variation in counted numbers among samples of any type collected at different locations and/or times. Overdispersion was therefore modeled as a Poisson–gamma mixture (Teunis, Rutjes, Westrell, & de Roda Husman, 2009). For a given set of $N_{\text{r}}$ replicate counts $k=\{k_{1},k_{2},\ldots k_{N\text{r}}\}$ in equivalent volumes $V=\{V_{1},V_{2},\ldots V_{N\text{r}}\}$ diluted from the same sample, the likelihood is Poisson

(1) $$\ell_{\text{s}}\left(c|k,V\right)=\prod_{i=1}^{N_{\text{r}}}\frac{{\left(cV_{i}\right)}^{k_{i}}}{k_{i}!}{\text{e}}^{-cV_{i}}$$

with concentration *c* and (equivalent) sample volume *V*. In case of surface swab or solid samples, these concentrations are converted to CFU per surface area or per gram, by using equivalent surface area $S_{i}$/weight $M_{i}$ instead of volume $V_{i}$. Assuming a gamma‐distributed concentration f(c|ρ,λ), the (marginal) likelihood for a set of $N_{\text{s}}$ samples {(***k***_1_, ***V***_1_), $\left(k_{2},V_{2}\right),\cdots ,\left(k_{N_{\text{s}}},V_{N_{\text{s}}}\right)\}$ is:

(2) $$\ell \left(\rho ,\lambda \right)=\prod_{j=1}^{N_{\text{s}}}\int_{c=0}^{\infty }f\left(c|\rho ,\lambda \right)\ell_{\text{s}}\left(c|k_{j},V_{j}\right)\text{d}c.$$

**Figure 2:**
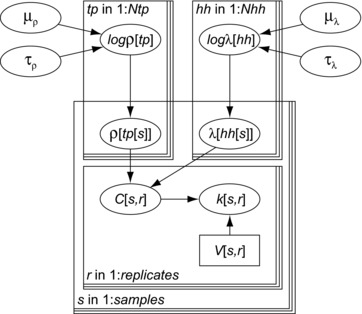
Directed acyclic graph (Gilks, Richardson, & Spiegelhalter, 1996) of the model for assessing variation in microbial concentrations. Replicate counts *k* in equivalent volume *V* of any replicate r=1,2,… of sample s=1,2,… are Poisson distributed with concentration *C*. That concentration is a random gamma variate with shape (clustering) parameter ρ and scale parameter λ. ρ is assumed to depend on sample type (*tp*), and λ is assumed to depend on household and/or neighborhood (*hh*). logρ and logλ are normally distributed with parameters μ and τ.

The parameters ρ and λ describe the variation in concentration among different samples. These two parameters may vary as well, for instance, by sample type or by neighborhood. It was assumed that the shape parameter ρ varied with the type of substrate, whereas the scale parameter λ changed with location. Therefore, variation among sample types was modeled as a distribution of ρ, whereas variation among households and/or neighborhoods was modeled as a distribution of λ. Fig. 2 shows the structure of this model and Table I details all parameters and distributions used.

**Table I:** Variables, Parameters, and Distributions Used in the Model

Variables	Parameters	Distributions/Models	Probability Density Function/Formulas	Sources
Area of the hand touching the surface (${\text{cm}}^{2}$)	μ=2.75	Log‐normal	$f\left(x\right)=\frac{1}{\sigma x\sqrt{2\pi }}e^{-{\left(\log\left(x\right)-\mu \right)}^{2}/2\sigma^{2}}$	USEPA (2011)
	σ=0.75			
Fraction of the surface of hands placed in mouth	α=3.7	Beta	$f\left(x\right)=\frac{x^{\alpha -1}{\left(1-x\right)}^{\beta -1}}{B(\alpha ,\beta )}$	Özkaynak et al. (2011); Zartarian et al. (2006)
	β=25			
Attachment coefficient from surfaces to hands	a=0.01	Triangle	$f\left(x\right)=\left\{\begin{array}{c} \frac{2(x-a)}{\left(b-a)(c-a\right)}\;\text{for}\;a\leq x<c\\ \frac{2(b-x)}{\left(b-a)(b-c\right)}\;\text{for}\;c\leq x\leq b\\ 0\;\;\;\;\;\;\;\text{otherwise} \end{array}\right.$	Özkaynak et al. (2011)
	b=0.03			
	c=0.02			
Detachment coefficient from hands to surfaces	a=0.5	Triangle	$f\left(x\right)=\left\{\begin{array}{c} \frac{2(x-a)}{\left(b-a)(c-a\right)}\;\text{for}\;a\leq x<c\\ \frac{2(b-x)}{\left(b-a)(b-c\right)}\;\text{for}\;c\leq x\leq b\\ 0\;\;\;\;\;\;\;\text{otherwise} \end{array}\right.$	Assumption
	b=0.95			
	c=0.75			
Duration of handwashing (seconds)	k=2.5	Gamma plus 10s (constant value)	$f\left(x\right)=\frac{1}{\Gamma \left(k\right)\theta^{k}}x^{k-1}e^{-x/\theta }$	Assumption
	θ=10			
Duration of bathing (seconds)	k=4	Gamma plus 60s (constant value)	$f\left(x\right)=\frac{1}{\Gamma \left(k\right)\theta^{k}}x^{k-1}e^{-x/\theta }$	Assumption
	θ=60			
Log10 fraction of microbes remaining after handwashing or bathing with soap	$\beta_{0}=2.18$	Linear model with log(duration)	$f\left(x\right)=\beta_{0}+\beta_{1}log\left(x\right)$	Montville, Chen, and Schaffner (2002); Kampf and Kramer (2004)
	$\beta_{1}=-1.18$			
Log10 fraction of microbes remaining after handwashing or bathing without soap	$\beta_{0}=0.68$	Linear model with log(duration)	$f\left(x\right)=\beta_{0}+\beta_{1}log\left(x\right)$	Montville et al. (2002); Kampf and Kramer (2004)
	$\beta_{1}=-0.80$			
Fraction of microbes removed from hands by hand–mouth contact	a=0.01	Triangle	$f\left(x\right)=\left\{\begin{array}{c} \frac{2(x-a)}{\left(b-a)(c-a\right)}\;\text{for}\;a\leq x<c\\ \frac{2(b-x)}{\left(b-a)(b-c\right)}\;\text{for}\;c\leq x\leq b\\ 0\;\;\;\;\;\;\;\text{otherwise} \end{array}\right.$	Amadi, Nwagu, and Emenuga (2013)
	b=0.40			
	c=0.33			
Probability of handwashing with soap	α=5	Beta	$f\left(x\right)=\frac{x^{\alpha -1}{\left(1-x\right)}^{\beta -1}}{B(\alpha ,\beta )}$	Estimate from structured observation data
	β=17			
Probability of bathing with soap	α=45	Beta	$f\left(x\right)=\frac{x^{\alpha -1}{\left(1-x\right)}^{\beta -1}}{B(\alpha ,\beta )}$	Estimate from structured observation data
	β=19			
Adherence coefficient of soil to hand (${\text{mg/cm}}^{2}$)	μ=0.11	Log‐normal	$f\left(x\right)=\frac{1}{\sigma x\sqrt{2\pi }}e^{-{\left(\log\left(x\right)-\mu \right)}^{2}/2\sigma^{2}}$	Özkaynak et al. (2011)
	σ=2.0			
Adherence coefficient of water to skin ($\mu {\text{L/cm}}^{2}$)	μ=5.4	Log‐normal	$f\left(x\right)=\frac{1}{\sigma x\sqrt{2\pi }}e^{-{\left(\log\left(x\right)-\mu \right)}^{2}/2\sigma^{2}}$	Gujral, Proctor, Su, and Fedoruk (2011)
	σ=0.5			
Frequency of hand–surface contact (per hour) for children	k=1.85	Weibull	$f\left(x\right)=\left\{\begin{array}{c} \frac{k}{\lambda }{\left(\frac{x}{\lambda }\right)}^{k-1}e^{{-(x/\lambda )}^{k}}\;x\geq 0\\ 0\;\;\;\;\;\;\;\;\;x<0 \end{array}\right.$	Freeman et al. (2001); Assumption
	λ=145			
Frequency of indoor hand–mouth contact (per hour) for children	k=0.91	Weibull	$f\left(x\right)=\left\{\begin{array}{c} \frac{k}{\lambda }{\left(\frac{x}{\lambda }\right)}^{k-1}e^{{-(x/\lambda )}^{k}}\;x\geq 0\\ 0\;\;\;\;\;\;\;\;\;x<0 \end{array}\right.$	Xue et al. (2007)
	λ=18.79			
Frequency of outdoor hand–mouth contact (per hour) for children	k=0.98	Weibull	$f\left(x\right)=\left\{\begin{array}{c} \frac{k}{\lambda }{\left(\frac{x}{\lambda }\right)}^{k-1}e^{{-(x/\lambda )}^{k}}\;x\geq 0\\ 0\;\;\;\;\;\;\;\;\;x<0 \end{array}\right.$	Xue et al. (2007)
	λ=13.76			
Probability of contact with own feces during defecation for age group two to five years	α=5	Beta	$f\left(x\right)=\frac{x^{\alpha -1}{\left(1-x\right)}^{\beta -1}}{B(\alpha ,\beta )}$	Estimate from structured observation data
	β=41			
Probability of hand–surface contact during defecation	α=24	Beta	$f\left(x\right)=\frac{x^{\alpha -1}{\left(1-x\right)}^{\beta -1}}{B(\alpha ,\beta )}$	Estimate from structured observation data
	β=18			
Frequency of hand–surface contact during defecation	a=1	Uniform	$f\left(x\right)=\left\{\begin{array}{c} \frac{1}{b-a}\;\text{for}\;x\in \left[a,b\right]\\ 0\;\;\;\text{otherwise} \end{array}\right.$	Assumption
	b=10			
Probability of exclusively breastfeeding (by age)	$P_{0-1}=0.339$	Constant	$P=\left\{\begin{array}{c} 0.339\;\text{for}\;\text{children}\;\text{0--1}\;\text{year}\;\text{old}\\ 0.007\;\text{for}\;\text{children}\;\text{1--2}\;\text{years}\;\text{old}\\ 0\;\;\;\;\;\text{for}\;\text{children}\;\text{2--5}\;\text{years}\;\text{old} \end{array}\right.$	Ghana Statistical Service (2016)
	$P_{1-2}=0.007$			
	$P_{2-5}=0$			
Probability of breastfeeding given not exclusively breastfed for children of age group zero to one year	α=79	Beta	$f\left(x\right)=\frac{x^{\alpha -1}{\left(1-x\right)}^{\beta -1}}{B(\alpha ,\beta )}$	Estimate from structured observation data
	β=46			
Probability of breastfeeding given not exclusively breastfed for children of age group one to two years	α=85	Beta	$f\left(x\right)=\frac{x^{\alpha -1}{\left(1-x\right)}^{\beta -1}}{B(\alpha ,\beta )}$	Estimate from structured observation data
	β=114			
Probability of breastfeeding given not exclusively breastfed for children of age group two to five years	α=10	Beta	$f\left(x\right)=\frac{x^{\alpha -1}{\left(1-x\right)}^{\beta -1}}{B(\alpha ,\beta )}$	Estimate from structured observation data
	β=90			
Probability of eating raw produce or bought food, given not breastfed, for children zero to one year old	α=31	Beta	$f\left(x\right)=\frac{x^{\alpha -1}{\left(1-x\right)}^{\beta -1}}{B(\alpha ,\beta )}$	Estimate from structured observation data
	β=16			
Probability of eating raw produce or bought food, given not breastfed, for children one to two years old	α=72	Beta	$f\left(x\right)=\frac{x^{\alpha -1}{\left(1-x\right)}^{\beta -1}}{B(\alpha ,\beta )}$	Estimate from structured observation data
	β=43			
Probability of eating raw produce or bought food, given not breastfed, for children two to five years old	α=74	Beta	$f\left(x\right)=\frac{x^{\alpha -1}{\left(1-x\right)}^{\beta -1}}{B(\alpha ,\beta )}$	Estimate from structured observation data
	β=17			
Probability of eating food with hands	α=254	Beta	$f\left(x\right)=\frac{x^{\alpha -1}{\left(1-x\right)}^{\beta -1}}{B(\alpha ,\beta )}$	Estimate from structured observation data
	β=46			
Serving weight for raw produce (g)	k=2.5	Gamma	$f\left(x\right)=\frac{1}{\Gamma \left(k\right)\theta^{k}}x^{k-1}e^{-x/\theta }$	Estimate from structured observation data and assumption
	θ=40			
Serving weight for prepared food and bought food (g)	μ=100	Normal	$P\left(x\right)=\frac{1}{\sigma \sqrt{2\pi }}e^{-{\left(x-\mu \right)}^{2}/2\sigma^{2}}$	Estimate from structured observation data and assumption
	σ=15			
Probability of using sachet water as drinking water in Alajo	α=154	Beta	$f\left(x\right)=\frac{x^{\alpha -1}{\left(1-x\right)}^{\beta -1}}{B(\alpha ,\beta )}$	Estimate from structured observation data
	β=47			
Probability of using sachet water as drinking water in Bukom	α=145	Beta	$f\left(x\right)=\frac{x^{\alpha -1}{\left(1-x\right)}^{\beta -1}}{B(\alpha ,\beta )}$	Estimate from structured observation data
	β=57			
Probability of using sachet water as drinking water in Old Fadama	α=187	Beta	$f\left(x\right)=\frac{x^{\alpha -1}{\left(1-x\right)}^{\beta -1}}{B(\alpha ,\beta )}$	Estimate from structured observation data
	β=14			
Probability of using sachet water as drinking water in Shiabu	α=151	Beta	$f\left(x\right)=\frac{x^{\alpha -1}{\left(1-x\right)}^{\beta -1}}{B(\alpha ,\beta )}$	Estimate from structured observation data
	β=49			
Daily consumption of tap water for age group zero to one year (cup)	μ=0.371	Log‐normal	$f\left(x\right)=\frac{1}{\sigma x\sqrt{2\pi }}e^{-{\left(\log\left(x\right)-\mu \right)}^{2}/2\sigma^{2}}$	Estimate from structured observation data
	σ=0.683			
Daily consumption of tap water for age group one to two years (cup)	μ=0.927	Log‐normal	$f\left(x\right)=\frac{1}{\sigma x\sqrt{2\pi }}e^{-{\left(\log\left(x\right)-\mu \right)}^{2}/2\sigma^{2}}$	Estimate from structured observation data
	σ=0.642			
Daily consumption of tap water for age group two to five years (cup)	μ=1.080	Log‐normal	$f\left(x\right)=\frac{1}{\sigma x\sqrt{2\pi }}e^{-{\left(\log\left(x\right)-\mu \right)}^{2}/2\sigma^{2}}$	Estimate from structured observation data
	σ=0.702			
Daily consumption of sachet water for age group zero to one year (sachet)	μ=0.040	Log‐normal	$f\left(x\right)=\frac{1}{\sigma x\sqrt{2\pi }}e^{-{\left(\log\left(x\right)-\mu \right)}^{2}/2\sigma^{2}}$	Estimate from structured observation data
	σ=0.631			
Daily consumption of sachet water for age group one to two years (sachet)	μ=0.597	Log‐normal	$f\left(x\right)=\frac{1}{\sigma x\sqrt{2\pi }}e^{-{\left(\log\left(x\right)-\mu \right)}^{2}/2\sigma^{2}}$	Estimate from structured observation data
	σ=0.677			
Daily consumption of sachet water for age group two to five years (sachet)	μ=1.060	Log‐normal	$f\left(x\right)=\frac{1}{\sigma x\sqrt{2\pi }}e^{-{\left(\log\left(x\right)-\mu \right)}^{2}/2\sigma^{2}}$	Estimate from structured observation data
	σ=0.478			
Duration of behavior (minutes)	k=4.48	Weibull	$f\left(x\right)=\left\{\begin{array}{c} \frac{k}{\lambda }{\left(\frac{x}{\lambda }\right)}^{k-1}e^{{-(x/\lambda )}^{k}}\;x\geq 0\\ 0\;\;\;\;\;\;\;\;\;x<0 \end{array}\right.$	Estimate from structured observation data & Teunis et al. (2016)
	λ varies by age group and neighborhood			
*E. coli* concentration (CFU/mg or CFU/mL or ${\text{CFU/cm}}^{2}$)	*k* varies by sample type	Gamma	$f\left(x\right)=\frac{1}{\Gamma \left(k\right)\theta^{k}}x^{k-1}e^{-x/\theta }$	Estimate from environmental sample data
	θ varies by sample type & neighborhood			

### 2.3. Exposure Factors

To calculate the numbers of microbes transferred through any of the contact behaviors with any environmental contamination additional data are needed. Questionnaire responses on water use, sanitation, and hygiene were collected in 800 households in four neighborhoods in Accra, Ghana. When possible, exposure factors were estimated from these survey data. Wherever such data were not available, the parameters were based on published scientific literature or assumptions, as noted below. This section describes additional modules used in the exposure model to specify exposure factors.

#### 2.3.1. Binary Attributes of Behavior

There are many Boolean variables describing whether behaviors have a certain attribute or not (see Table I), for example, handwashing with versus without soap, or eating with hands versus using cutlery. In such cases, the probability of either choice was estimated from structured observation or survey data, by assuming a binomial likelihood

(3) P(y|θ)=nyθy(1−θ)n−y

and an uninformed uniform prior distribution for the probability θ∼Beta(1,1), leading to a Beta distributed posterior

(4) $$P\left(\theta |y\right)\propto \theta^{y}{\left(1-\theta \right)}^{n-y}$$

with parameters θ|y∼Beta(y+1,n−y+1).

#### 2.3.2. Water Consumption

The variables describing consumption of water, including tap water and sachet water, were estimated using the household survey data, where respondents stated how many cups/sachets of water they consumed daily. A single cup was assumed to measure 237 mL, and a sachet was assumed to contain 500 mL of water (Kwakye‐Nuako, Borketey, Mensah‐Attipoe, Asmah, & Ayeh‐Kumi, 2007; Stoler, Weeks, & Otoo, 2013). As water intake may be any positive real number, the responses were treated as interval‐censored data. If the individual daily water intake is log‐normal with parameters μ and σ, then the likelihood function

(5) $$\begin{array}{c} \ell \left(\mu ,\sigma |y\right)=\prod_{i=1}^{n}\left[\Phi \left(\frac{\text{log}(U_{i})-\mu }{\sigma }\right)-\Phi \left(\frac{\text{log}(L_{i})-\mu }{\sigma }\right)\right] \end{array}$$

may be used, where observation *i* consists of the interval ($L_{i},U_{i}$), and Φ is the cumulative distribution function (CDF) of the normal distribution. Drinking water consumption was stratified by age groups but assumed to not vary between neighborhoods.

#### 2.3.3. Transfer of Microbes by Hand Contact

When a hand makes contact with a contaminated surface, any microbe on the contacted surface may be transferred to the hand. If the transfer probability is equal for all microbes, the eventual number transferred to the hand *m* is a binomial sample of the total number present on the contacted surface *M*.

Alternatively, when a contaminated hand touches a surface, some of the attached microbes will be transferred from the hand to the contacted surface. In this case, the probability of transfer is again fixed, the number of microbes transferred from the hand to the surface *n* is again a binomial sample of the total number that was present on the hand contact area *N*.

DetachmentN→N−nNnpn(1−p)N−nAttachmentN→N+mMmqm(1−q)M−m

*N* is a proportion of the total number of microbes on the hands (*NH*) and depends on the area of hands touching a contaminated surface. These two events—attachment and detachment of microbes (or particles containing microbes)—happen simultaneously, so that the probability that a number k=N−n+m remains on the hands is:

(6) P(k)=∑n=0NNnpn(1−p)N−nMn−N+k×qn−N+k(1−q)M−n+N−k,

which can be recursively applied to calculate the distribution of microbes on hands after touching a surface 1, 2, … times. When repeatedly touching the same surface, the expected numbers of microbes may be easily calculated using:

(7) $$\left(\begin{array}{c} S_{n+1}\\ H_{n+1} \end{array}\right)=\left(\begin{array}{cc} 1-p & q\\ p & 1-q \end{array}\right)\left(\begin{array}{c} S_{n}\\ H_{n} \end{array}\right).$$

The expected numbers of microbes on hands *H* and on surface *S* will approach a steady state ($H_{n}\approx H_{n+1},S_{n}\approx S_{n+1}$). The number of touching events needed to reach a steady state depends on the attachment and detachment coefficients *p* and *q*. As the sum of the coefficients p+q approaches 1, a steady state is reached in a single event; when p+q=0.63, it takes three touching events to arrive within 5% range of the steady state. As the fractions p+q observed tend to be high (Julian, Leckie, & Boehm, 2010; Mackintosh & Hoffman, 1984; Rusin, Maxwell, & Gerba, 2001), the parameters used in this study were set to reach a steady state within a few touching events.

Modifications to the parameters for contact with fomites, drains, direct contact with (own) feces, and intake of microbes when mouthing contaminated hands are described in Section 3.1. Details of parameter settings are listed in Table I.

## 3. MODEL DEVELOPMENT AND IMPLEMENTATION

The simulation model describes a dynamic network structure (Fig. 1), tracking microbes from different sources in the environment through different pathways to human ingestion. At the beginning of a day, an initial state is assumed (i.e., sleeping in bed, “off‐ground”). The behavioral simulation model generates a next state (behavior and compartment) and a time when transition to the next state occurs. Depending on the state, certain types of contacts may occur with a specific environmental compartment. For each type of contact, there are corresponding exposure factors and a level of contamination (i.e., fecal microbe concentration). For example, if the state is playing/sitting at an open drain, then the linked contaminant level is the concentration of microbes in drain water, and the exposure factor is the coefficient of attachment from drain water to hands. The numbers of microbes transferred during any state are simulated in exposure modules, using the environmental contamination level, the current contamination level on hands, and the exposure factors as inputs. There is an exposure module for any type of contact, defined by any state in the behavioral simulation model.

After calculation of the numbers of microbes transferred between current nodes in the network and updating the numbers ingested, the simulation moves to the next state with a corresponding duration. Thus, successive updates of the numbers of microbes on hands and the numbers ingested are generated at random intervals as determined by the behavioral simulation model (Fig. 1). The simulation is continued until a defined total duration has elapsed, usually a daytime period of 14 hours. Then, a new sequence is started, representing a new individual and generating a different behavior sequence with its exposure profile until the required population size is reached, usually 10,000 individuals.

As the transfers of each individual microbe within the network are known during the entire simulation, any single microbe can be traced from its source to any sink (ingestion or removal from hands by handwashing or bathing). This allows calculation of the contribution of any source or even any pathway (i.e., path through the network) of human exposure. Observations of behavior and microbial contamination are thus combined to trace movement of microbes that cannot be directly observed.

### 3.1. Module Development

Seven different modules (submodels) were created to calculate numbers of microbes transferred between *sources*, *vehicles*, *sinks*, and *ingestion* (a special sink where exposure occurs). These modules were applied according to the “state” of the simulated subject as illustrated in Fig. 1 (compartment and behavior combination; see Table II). The details of all module functions are listed below:

**hand.fomites and hand.mouthing**. Fig. 1 shows how the hand.fomites module and hand.mouthing calculates numbers of microbes transferred during playing/sitting behavior. During playing/sitting behavior, hand‐to‐fomite contact and hand‐to‐mouth contact occur independently and repeatedly. Each contact updates the numbers of microbes on hands (*NH*) and numbers of microbes ingested (*NI*) with the corresponding function. For hand‐to‐fomite contact, the numbers of fecal microbes attached to, and detached from, hands are both assumed to be binomial (Section 2.3.3). The area of hands touching the surface determines the proportion of fecal microbes on hand and the number of fecal microbes on the contaminated surfaces are involved in the binomial process as *N* and *M*. For hand‐to‐mouth contact, we assume that only detachment from hands occurs. As a result, during playing/sitting behavior, the numbers of microbes on hands may increase or decrease while the ingestion accumulates. During sleeping behavior, only hand.mouthing is applied.
**hand.drain**. This module quantifies the microbe transfer from highly contaminated drain water to hands during the state “playing/sitting at drain.” And the detachment of microbes from hands was assumed to be negligible.
**hand.defecation**. For defecation behavior, all children may touch their own feces with a small probability. With such a contact, a high number of microbes may be attached to the hands and detachment is likely to be negligible. Only children in the two‐ to five‐year‐old age group were assumed to have hand‐to‐fomite contacts with the current compartment in the hand.fomites module.
**hand.washing/hand.bathing**. These modules simulate reduction in the numbers of microbes on hands due to washing or bathing. Detachment is assumed to be a binomial random number between 0 and the number of microbes currently on hands. The detachment coefficient depends on the duration of handwashing or bathing. Attachment of microbes from water to hands was assumed to be negligible.
**eating**. The eating module calculates the numbers of microbes ingested with food, which depends on food type. For breastfeeding, there is no ingestion of microbes since breast milk is assumed to be free of fecal microbes. Contamination of the breast skin was not accounted for because it was difficult to obtain specific data on skin contamination.
  If children eat produce that is typically consumed without cooking (e.g., lettuce, tomatoes, cucumbers), it is assumed that the outer surface of the raw produce items is touched first, before contact with the inner (edible) parts. Thus, handling of the food is simulated (microbe transfer between food and hands) during eating behavior. Such an order of events tends to contaminate the inner parts, which are assumed to have been clean before that contact event. If the food type is “prepared food” or “vended food” and it is eaten by hands, then the module assumes that a single hand–food contact was made before ingestion.
**drinking**. The drinking module determines how much water children drink during a 14‐hour daytime period, and which type of water is chosen (tap water or sachet water).

**Table II:** Exposure Pathways, Links to Behaviors and to Environment Type/Sample‐Type Combinations for Neighborhoods: 1 = Alajo, 2 = Bukom, 3 = Old Fadama, 4 = Shiabu

Compartment	Behavior	Module
Dirt floor	Playing/sitting	hand.fomites
		hand.mouthing
Concrete floor	Playing/sitting	hand.fomites
		hand.mouthing
Off‐ground	Playing/sitting	hand.fomites
		hand.mouthing
Stagnant water and trash area/drain	Playing/sitting	hand.drain
–	Sleeping	hand.mouthing
–	Handwashing	hand.washing
–	Bathing	hand.bathing
–	Defecating	hand.defecation
		hand.fomites
–	Eating produce	eating
–	Eating prepared/bought food	eating
–	Drinking tap water	expos.dw
–	Drinking sachet water	expos.dw

Fig. 1 shows how, during any hand–surface contact and hand–mouthing contact, the number of transferred microbes depends on the area of the hand touching the surface, the fraction of the hand placed in the mouth, the contamination level on the surfaces, the numbers of microbes on the hands, and the coefficients of attachment and detachment. The updated numbers of microbes on the hands and the numbers ingested also depend on additional information, including the frequency of touching the surface, the frequency of mouthing, and the order in which those contacts occur. Probability distributions were defined for all these additional factors (Table I).

### 3.2. Model Implementation

Parameter estimation was implemented using a Bayesian framework coded in JAGS (v4.1.0) (Plummer, 2003) using rjags (v4‐6) (Plummer, 2013) within R (v3.2.4) (R Core Team, 2015), with carefully chosen priors, uninformed where possible (Table I).

**Figure 3:**
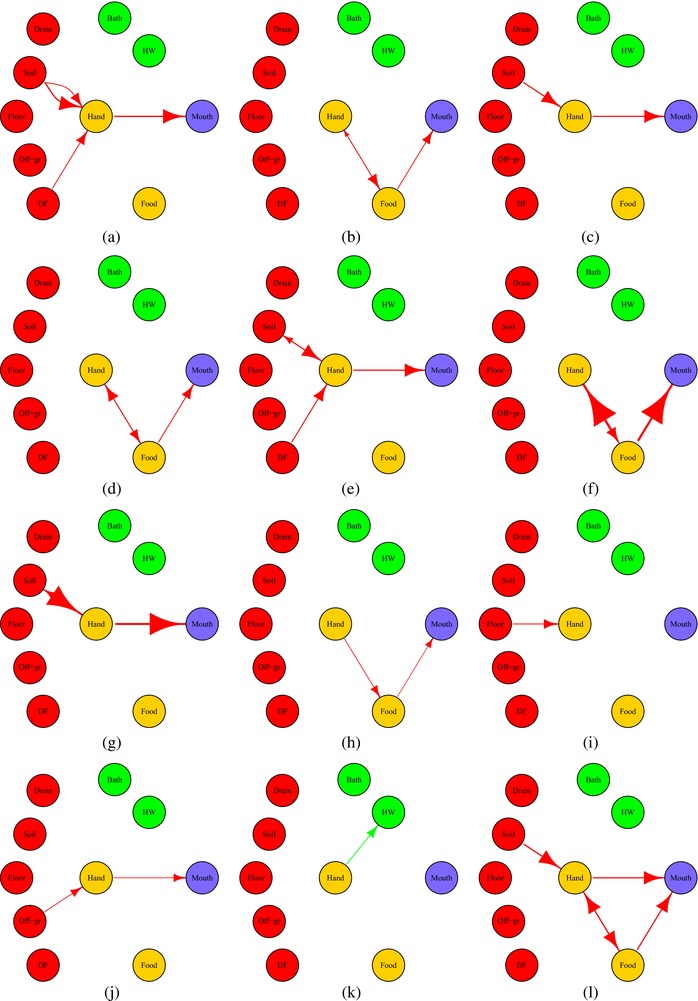
Snapshots (along with time, order from figure (a) to figure (l)) of fecal microbe transfer network for a typical child day (two to five years old, Bukom neighborhood). DF = “direct contact with own feces,” HW = “handwashing.” The weights of edges are proportional to the log10 number of microbes transferred. The color of nodes represents their role in the network. Red: sources; yellow: vehicles (can be source and sink); green: sinks (remove contamination); blue: ingestion.

Exposure simulations were run in R (v3.2.4) (R Core Team, 2015). Typically, 14‐hour simulations were simulated for 10,000 subjects, stratified by age group and neighborhood. The output of the simulations consisted of numbers of microbes transferred between any sources and sinks in the model (Fig. 3).

Model output can be summarized as distributions describing daily exposure from different pathways, for each neighborhood and age group (Wang et al., 2017). The outputs can also be viewed as dynamic transfers within a network structure (Fig. 3).

### 3.3. Simplifying Assumptions

In order to keep the model manageable, simplifying assumptions had to be made, especially where there were knowledge gaps. Key assumptions are listed below to clearly define the constraints of the model.

1. All simulated child‐days started with the state “sleeping off‐ground,” and the length of a child‐day (daytime period) was exactly 14 hours.
2. The coefficient of detachment was high, and larger than the coefficient of attachment, so that repeated touching of a contaminated surface rapidly resulted in stable numbers of microbes on hands. This is consistent with published studies on hand contamination that examined effects of repeated touching (Gibson, Rose, Haas, Gerba, & Rusin, 2002; Nicas & Jones, 2009).
3. The die‐off rate of microbes was set to zero, which means that the number of viable microbes was not assumed to decrease during the time they were attached to hands.
4. Only part of a hand has contact with fomites and enters the mouth during hand mouthing. It was assumed that all microbes present on hands after a contact event were instantaneously redistributed on the skin, so that their surface density remained uniform.
5. In case raw produce or fruits were consumed using hands, it was assumed that the hands touch the (potentially contaminated) outer skin of the produce first and then contact the edible parts, for instance, by peeling off the skin. The inner parts were assumed to have been clean before breaking the skin.
6. It was assumed that breast milk was not contaminated and that no microbes were indirectly ingested through skin contact during breastfeeding.
7. No hand mouthing was assumed to occur during defecation, but hands may inadvertently touch feces (with low probability) or contaminated surfaces during defecation.
8. No hand contact with any contaminated surfaces was assumed to occur during sleep, but hand mouthing may occur.

## 4. RESULTS

### 4.1. Sources of Randomness

**Figure 4:**
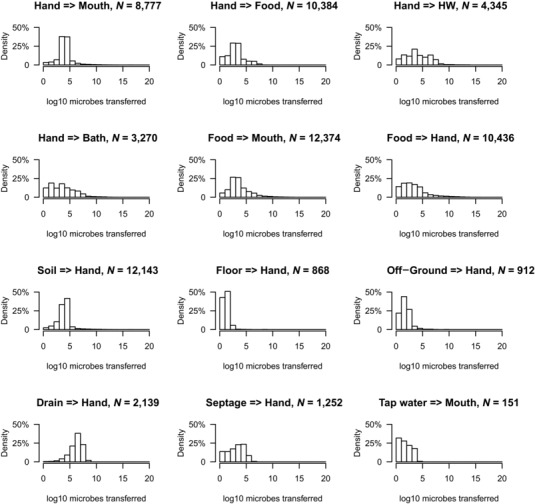
Histograms of numbers of microbes transferred between different nodes in the fecal microbe transfer network for two to five year olds in the Bukom neighborhood. *N* represents the total number of a specific type of transfer occurring in 10,000 simulated child‐days of 14 hours. offgr = “off‐ground surfaces,” DF = “direct contact with own feces,” HW = “handwashing,” Bath=“bathing.”

The multipathway exposure assessment model was applied to data from four neighborhoods in Accra, Ghana, to assess the exposure to fecal contamination for children under 5 living in those neighborhoods. Simulated microbe transfers were generated to quantify exposures from different sources. As environmental microbe concentration, length of contact, and transfer coefficients (directional) are all random variables defined by a distribution, each microbe transfer between two nodes (including sources, hands, and sinks) will show random variation. For example, two events of touching a dirt floor lead to different numbers of microbes transferred from soil to the hands. The concentration of microbes in dirt, the contacted area, and the transfer coefficient are all random variables. Fig. 4 shows histograms of simulated numbers of microbes transferred between different nodes for 10,000 simulated (14‐hour) child‐days in the Bukom neighborhood.

**Figure 5:**
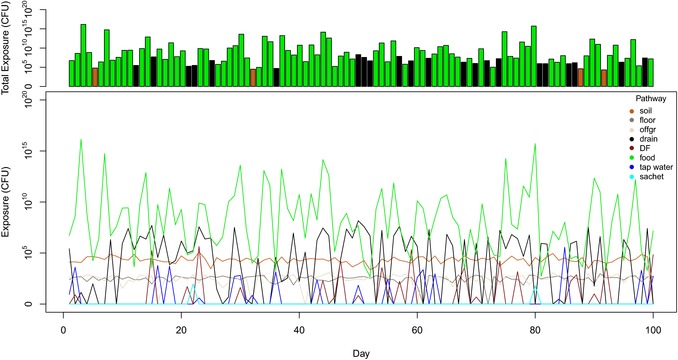
Time course of exposure to *E. coli* (CFU) by pathway and dominant pathway for 100 simulated days. Bottom graph: time course of exposure by pathway for 100 simulated days. Top graph: bar chart for total exposure, the color of each bar represents the dominant pathway for that day. offgr = “off‐ground surfaces,” DF = “direct contact with own feces,” sachet = “sachet water.”

Fig. 5 shows a simulated time course of the contributions to exposure from different sources of fecal contamination, averaged over 100 simulated days. Food (10^2.68^–10^16.15^ CFU/day) and open drains (10^0.48^–10^8.17^ CFU/day) are the sources that provide the greatest contribution to exposure, but these are also the most variable sources of exposure to fecal contamination. Dirt floor (soil) is also a major source (10^3.36^–10^5.10^ CFU/day), but its contribution is less variable.

Comparison of exposure by neighborhood and age is shown in a companion paper (Wang et al., 2017); see Figs. 3 and 4, and details in Figs. A1–A7 in that paper. Exposure from food and soil occurred in any simulated child‐days, resulting in median total exposure of 10^6.34^ (95 percentile range: 10^3.32^–10^12.87^ CFU/child‐day) for food and 10^4.95^ (95 percentile range: 10^4.44^–10^5.39^ CFU/child‐day) for soil in Bukom and two‐ to five‐year‐old age group. Exposure via open drains and direct contact with (own) feces did not occur on all child‐days, and resulted in somewhat lower exposures: 10^5.71^ (95 percentile range: 10^0.78^–10^7.37^ CFU/child‐day) and 10^2.95^ (95 percentile range: 0–10^5.06^ CFU/child‐day), respectively. Tap water, (concrete) floors, and off‐ground surfaces contributed much less to exposure. Different neighborhoods and age groups had similar outcomes, though exposure was highest in Shiabu (Wang et al., 2017).

### 4.2. Flows of Microbes

The dynamic nature of exposure to fecal contamination can be seen clearly in the network graphs of exposure pathways. Fig. 3 shows a series of successive snapshots of the flow of microbes in the network from sources (red) to sinks (green). Food (yellow) can be both a source of exposure and a sink, receiving fecal contamination from hands (yellow). In Fig. 3(a), a child defecated on the dirt floor and continued to sit and play on the dirt floor. A fraction of the fecal microbes he/she picked up from the dirt and his/her own feces were ingested. Then, in Fig. 3(b), the child ate raw produce using his/her hands, through which some of the fecal microbes on the hands were transferred to the food and ingested. By following these sequences of fecal microbe transfer, we can trace how microbes moved from the environment to human ingestion and quantify the numbers transferred. In this network of fecal pathways, the hands play a central role: they are the hub through which contamination is transferred to the mouth (blue).

Over a total of 10,000 simulated child‐days, the fraction direct exposure (i.e., not involving prior hand contact) is very high 0.86–0.99 for any exposure, for all ages and neighborhoods, and 0.69–1.00 if only food is considered. However, this fraction is highly variable as becomes apparent when calculating the same fraction for yearly exposure of a single child (365 child‐days) and averaging over 1,000 children: in zero to one year old for exposure to any source the mean fraction direct exposure is low, 0.2–0.4 (different neighborhoods), but with a (95%) range from 0 to 1. The median fraction is small (0.002–0.20), indicating a strongly skewed distribution. In older children (one to two years old and two to five years old), the mean fraction direct exposure is higher (0.5–0.7) but with the same range of variation (0–1).

### 4.3. Model Validation: Hand Contamination

To study whether the model assumptions were reasonable and model predictions were realistic, we conducted a validation experiment using hand rinse data collected during the SaniPath study. A total of 100 hand rinse samples for children under 5 were collected in the four study neighborhoods (Wang et al., 2017).

Because the simulation model allows us to quantitatively track microbes from any source to ingestion, the numbers of fecal microbes attached to hands at any time during a simulated daytime period are known. For each of 10,000 simulated child‐days in any age group, the numbers of fecal microbes on hands were sampled at a random time point. Thus, a cross‐sectional hand rinse study could be simulated, with a distribution of numbers of microbes on hands in any neighborhood and age group. Note that the model calculates the numbers of microbes on hands from environmental sources (Fig. 3), not from hand rinse data.

**Figure 6:**
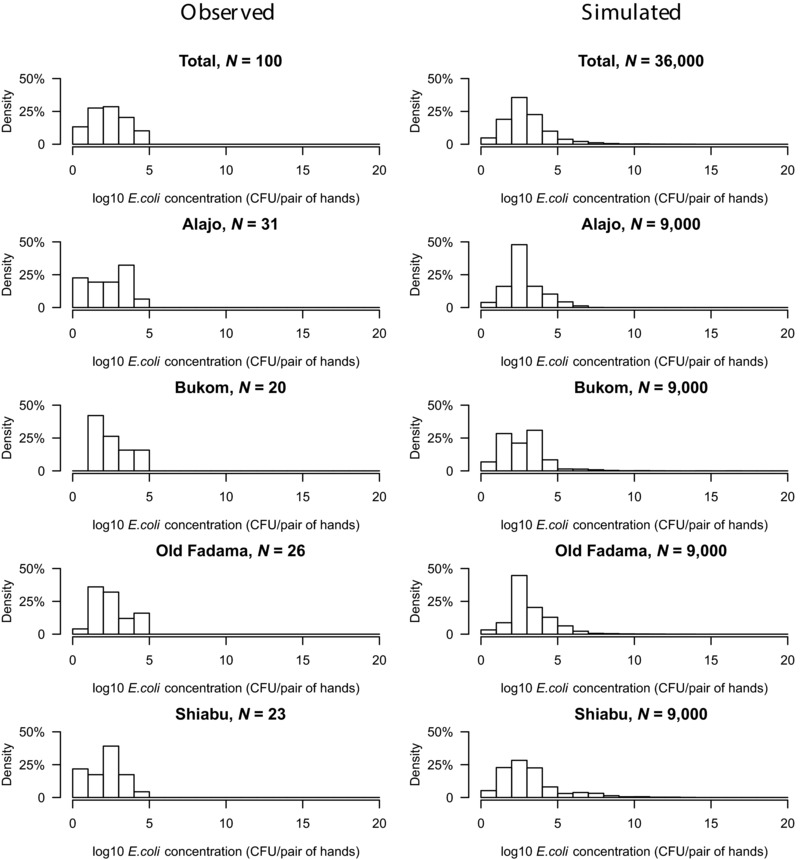
Histograms of *E. coli* hand contamination from analyses of hand rinses (observed) and from simulations for children under 5. The histograms in the left column are from observation. The upper limit of detection (ULOD) is 10^5^ CFU/pair of hands. The histograms in the right column are from simulation. Three thousand iterations were simulated for each age group and neighborhood, resulting in 9,000 iterations for each neighborhood and 36,000 iterations in total.

The hand contamination predicted by the model was then compared to the observed numbers of microbes in the hand rinse samples from the study neighborhoods. Fig. 6 shows numbers of fecal microbes (*E. coli*) on hands as inferred from hand rinse samples, to compare with the simulated hand contamination data. The magnitudes of *E. coli* concentration in the simulated results and observed data are similar. Because there is an upper limit of detection of membrane filtration, the observed concentrations were truncated at 10^5^ CFU per pair of hands.

## 5. DISCUSSION

The exposure model presented here is based on a detailed model of human behavior, capable of generating sequences of activities in a specific order. Aside from frequencies of activities or their duration, the order in which these activities occur determines their effect on exposure. This becomes clear when using network statistics to compare the fitted behaviors to models without preferential order (Teunis et al., 2016). The simulated behaviors are highly variable, but through simulation of a population, statistics (e.g., mean, standard deviation, or range) of the outcomes may be analyzed. This model cannot be directly verified by observations. However, the extent to which some model outputs compare with measured parameters may be examined, as illustrated for the hand contamination estimates.

In studies of microbial risk, exposure assessments commonly account for a single chain of processes (or several process chains), using deterministic (Labite et al., 2010; Machdar et al., 2013) or stochastic methods (Barker et al., 2014; McBride et al., 2013). In food QMRA, a general framework has been developed to investigate how microbes propagate on foods (Nauta, 2008), including the complexity due to variable human behavior during food preparation (Mylius, Nauta, & Havelaar, 2007). The present study introduces a new, interdisciplinary approach by integrating multipathway exposure assessments with social science methods (Ettema, Borgers, & Timmermans, 1995; Leszczyc & Timmermans, 2002) that provide a quantitative model of human behavior based on observational data. This comprehensive exposure model allows tracking of fecal microbe transfer throughout all modeled compartments, so that the fate of fecal contamination from any source may be determined. The combined influence of variable concentrations of microbes in environmental sources, variable intake of contaminated media, and variable exposure behavior causes a high degree of random variation. Nevertheless, food clearly dominated as a source of exposure to fecal contamination.

The development of the exposure model for the Sanipath study was a complex, in‐depth project that combined the work of a multidisciplinary research team with graduate‐level training in environmental microbiology, behavioral research, database building and management, biostatistics, programming, and project management. Specific components of this study (behavioral analyses, microbiological analyses) provide data that may be valuable for other risk models and studies. These data will become publicly available at the conclusion of the project. The source code of all models can be found on Github (Source code on Github, 2018). We have used the results and experience from this study to design a streamlined exposure assessment tool (Sanipath website, 2018) that can be used by a wide range of stakeholders to estimate risk of exposure to fecal contamination via different environmental pathways and use this information to guide decisions about investments in infrastructure (water, sanitation, and drainage) and agricultural practices.

### 5.1. Model Mechanisms

The exposure model includes two mechanisms of oral ingestion: direct and indirect. Direct ingestion represents eating contaminated food and drinking contaminated water, which are described in the eating and drinking modules. Indirect ingestion involves hands as a vehicle that connects contaminated fomites with ingestion. Touching fomites (food, drains, soil, floor, and off‐ground surfaces) enables microbes to transfer and attach to the hands, while hand mouthing leads to oral ingestion. Though microbes can attach to hands each time fomites are touched, hands will not accumulate contamination during repeated touching events because of detachment. Any microbes that attach upon touching may easily be lost during subsequent touching events or due to hygiene behavior (hand washing and bathing) that removes microbes attached to the hands.

### 5.2. Patterns of Variation in Exposure

The model captured two types of exposure: event‐like (Teunis et al., 2010) exposure (exposure associated with a relatively rare event), and low‐level background exposure. Event‐like exposure usually originates from a highly contaminated source (open drain or highly contaminated food) with large variation in contamination level. However, contact with such sources is rare, resulting in few exposures.

The low‐level background exposure pattern is associated with frequent contact with contaminated surfaces (soil, floor, or off‐ground surfaces). Another low‐intensity background exposure is associated with daily consumption of drinking water with low concentration of contamination.

In addition to the large variation in environmental contamination levels, other exposure factors may contribute to the variation of event‐like exposure. For the food pathway, breastfeeding behavior, food choice (raw produce, prepared food, ready‐to‐eat food), and food handling (using bare hands or cutlery) will add to the variation in foodborne exposure. Because of the variation in source concentrations and contact frequencies, event‐like exposure can show large differences between days (Fig. 5). In contrast, the contamination levels on surfaces in the private domain (i.e., household) plus frequent contacts with those surfaces lead to a stable level of exposure across successive days.

The main sources of variation in numbers of microbes transferred between sources, vehicles, and sinks are the variation in environmental contamination levels and the rapid and frequent changes in hand contamination during the day.

Chronic exposure to fecal contamination may lead to long‐term adverse health outcomes like environmental enteric dysfunction and stunting (Humphrey, 2009; Jiang et al., 2017; Mbuya & Humphrey, 2016). In intervention studies, it could be helpful to examine exposure patterns and compare frequent event‐like exposures (which may cause symptomatic infections due to a high dose) with sustained low‐level exposures (which may cause frequent, but asymptomatic, intestinal infections). This approach may help predict the effect of interventions aimed at avoiding event‐like exposures (e.g., covering open drains) and interventions that decrease chronic exposure to sustained low‐level contamination (e.g., improved water quality) on developing long‐term adverse health outcomes.

### 5.3. Dynamic Fecal Microbe Transfer Network

The F‐diagram (Wagner & Lanoix, 1958) illustrates how multiple pathways determine exposure to fecal microbes. The exposure model presented here extends this by describing a dynamic network of fecal microbe transfer and fate over time (Fig. 3). Because indirect ingestion is a stepwise process, the microbes transferred to hands by a single contact with fomites may require multiple handwashing events, touching other surfaces, and mouthing of the hand to disperse. Attachment and detachment occur during discrete events when hands are in contact with surfaces or fluids. As a consequence, a complete indirect ingestion pathway usually overlaps with other indirect ingestion pathways, influencing the fraction ingested from other sources. This complex interdependence of microbe transfers emphasizes how the order in which events occur is important.

### 5.4. Application of the Model to Inform Interventions

The main goal of this exposure model was to allow comparisons between exposures to fecal microbes via different pathways in order to provide guidance on where to effectively target interventions to achieve maximum reduction in overall exposure to fecal microbes. We would expect reductions in exposure to result in health benefits, but that may depend on the magnitude of the reduction in overall exposure to fecal microbes. By applying this exposure model, we can predict the reduction in ingestion of fecal microbes that would result from interventions targeted at a specific pathway or set of pathways. The preintervention exposure can be compared to the expected postintervention exposure to determine whether the ingestion of fecal microbes would be substantially reduced, presumed sufficient to result in health benefits (Briscoe, 1984).

Interventions may be infrastructure changes, such as reduction of environmental contamination (e.g., through the installation of sanitation systems that safely contain excreta), or preventing access to sources of contamination (e.g., through covering open drains). Alternatively, interventions may be behavioral, such as adjusting the time spent in different contaminated environments, or changing the frequency of event‐like behavior (high‐risk or hygiene) by reinforcing specific behaviors (e.g., handwashing before eating).

Any such intervention may be translated into a scenario by modifying some of the settings in the exposure model to simulate the effects of the intervention and predict the impact on ingestion of fecal microbes and possible reduction of enteric infections and associated adverse health outcomes.

Sensitivity analyses of the environmental and behavioral variables included in the exposure model allow identification of critical points (e.g., the dominant pathway, key exposure factors) that are major factors in controlling daily exposure. Among those critical factors, some may be control points, i.e., they can be manipulated to effectively reduce exposure. Identification of critical control points provides guidance for designing interventions that may most efficiently reduce exposure to fecal contamination.

The current model has multiple modules with hundreds of variables and parameters. In particular, modification of behavior sequences creates many different scenarios (e.g., enforcing handwashing before eating) that must be compared. Therefore, adding a comprehensive sensitivity analysis requires a substantial amount of text, and cannot be added to the current article. A separate paper will be dedicated to combined sensitivity analysis and simulated intervention scenarios.

### 5.5. Strengths and Limitations

This article describes a quantitative multipathway network structure model, which is novel in microbial exposure assessment. The model allows the comparison of different exposure pathways by standardizing the definition of exposure to fecal contamination as ingestion of CFU of *E. coli*. It also enables simulation‐based evaluation of infrastructure and behavior interventions. The dynamic fecal microbe transfer feature in this model extends the F‐diagram by showing how different barriers are dynamically modified by behavior patterns.

The choice to stop at exposure and not address infection and symptomatic illness endpoints was made at the very conception of the Sanipath project (Robb et al., 2017). As argued in (more) detail there, this choice was made with reference to Briscoe (1984), who argued that (observed) cases may not reflect the effects of a health intervention due to saturation of the dose–response relation at high exposures.

As in any modeling effort, there were several information gaps that required us to make assumptions, some of which were arbitrary. Additional information is needed about food consumption patterns, drinking behavior, and exposure factors like the frequency of touching different surfaces and the frequency of hand mouthing while playing for children under five years old.

The model aims to estimate human ingestion of fecal microbes from the environment. The current study only used *E. coli* to model enteric pathogens associated with fecal contamination. However, other fecal microbes could be plugged into this model to conduct exposure assessment. Follow‐up studies could examine other fecal indicators, or possibly human pathogens, which may have different transfer properties and environmental persistence.

Finally (and most ambitiously), data collection could be repeated in a different low‐income urban setting. The present study used data from four neighborhoods with varying income status, geographical location, and sanitary infrastructure, but was limited to a single city (Accra, Ghana). Repeating the same study design in a different city with a different geographic and cultural conditions could provide valuable insight into how much the results of the present study may be generalized to other cultures and climates.

## 6. CONCLUSION

This article describes a dynamic, multipathway exposure assessment model for children under five years of age in crowded, highly contaminated urban environments. Models of varying environmental concentrations of fecal microbes, child behaviors, and a comprehensive set of exposure factors were combined to simulate microbe transfer in a network structure from environmental sources to human ingestion. The results quantify variation in exposure within a specific fecal‐oral pathway and between pathways. The network structure representation highlights the importance of hands in transferring fecal microbes from the environment to the child's mouth. The output of the model can demonstrate the importance of different exposure pathways and thus help predict potential reductions in exposure by specific intervention scenarios. Further study of exposure factors and additional data collection are needed to fill in information gaps and improve model assumptions.
